# The Effect of Dairy Products and Nutrient Intake after Childbirth on the Risk of Postpartum Depression

**DOI:** 10.3390/ijerph192416624

**Published:** 2022-12-10

**Authors:** Shoug Alashmali, Arwa S. Almasaudi, Haya S. Zedan, Baian A. Baattaiah, Yazed Alashmali

**Affiliations:** 1 Department of Clinical Nutrition, Faculty of Applied Medical Sciences, King Abdulaziz University, Jeddah 21589, Saudi Arabia; 2 Department of Public Health, College of Health Sciences, Saudi Electronic University, Riyadh 13316, Saudi Arabia; 3 Department of Physical Therapy, Faculty of Medical Rehabilitation Sciences, King Abdulaziz University, Jeddah 21589, Saudi Arabia; 4 Ministry of Health, Jeddah 21432, Saudi Arabia

**Keywords:** dairy, nutrition, postpartum, depression, childbirth

## Abstract

Previous studies have shown an association between the intake of dairy products during pregnancy and reduced symptoms of postpartum depression (PPD). However, the effect of postpartum intake of dairy products on PPD is not fully understood. This study evaluates the effects of dairy products and nutrient intake after childbirth on the risk of PPD. A cross-sectional survey-based study was conducted asking participants to fill out a food frequency questionnaire (FFQ) to assess intake of dairy products and other nutrients. The Edinburgh Postnatal Depression Scale (EPDS) was used to screen for PPD symptoms. Out of 530 participants, almost three-quarters subjectively reported PPD (N = 395, 74.11%). The risk of PPD was relatively high for a Q1 level of consumption of all four dairy products and other nutrients, and from Q2 to Q4 there appeared to be an increase in the risk of PPD as consumption increased. However, after adjustment for confounding factors, there was no significant association between postpartum intake of dairy products and other nutrients and PPD. The results indicate that the potential of dairy products and nutrient intake to reduce PPD are minimal. Further longitudinal and intervention studies of dairy products and other (particularly anti-depressants) nutrients are required to draw firm conclusions about their associations with the risk of PPD.

## 1. Introduction

As an increasingly recognized complication of childbirth, postpartum depression (PPD) involves the appearance of symptoms of depression during the postpartum period, with onset generally between two and twelve weeks after delivery [1]. PPD is different from puerperal blues or maternity blues, which appear in most women and resolve before two weeks after birth, since PPD may last up to one year [2]. PPD is characterized by a depressed mood, excessive anxiety, insomnia, fatigue, and poor concentration [3]. Globally, the prevalence of PPD has increased in recent years, requiring attention and efforts from the medical community and policymakers to mitigate its short- and long-term effects. Research suggests that postpartum women who suffer from PPD are estimated to be between 5% and 60% worldwide [4,5]. In Saudi Arabia, a study by Alamoudi et al. (2017) found a PPD prevalence rate of over 50% [6]. The prevalence rate may be underreported in these published studies because many women may struggle with PPD but do not report their struggles to healthcare providers due to social and cultural barriers, and concerns around the stigma of mental health issues [7]. The World Health Organization (WHO) has issued recommendations on the urgent need to recognize and manage maternal mental health issues as a priority public health issue, as the consequences of PPD can have significant public health ramifications if left undiagnosed and untreated [8].

There is considerable debate around the safety of psychotropic medication during and after pregnancy [9,10]. Despite many studies on the safety and efficacy of pharmacological interventions for treating PPD [9,10], concerns remain about the potential adverse effects of certain types of pharmacological therapy on breastfed newborns and babies [10]. 

Alternative non-pharmacological interventions for treating depression in the postpartum period have not been widely investigated or well addressed. There have been several studies on the association between food groups and depression [11,12,13]. While several foods and nutrients are considered potential non-pharmacological management options for depression, there is a limited evidence base for non-pharmacological treatment options for this condition [14,15].

Dairy products are one of the main food groups that provide the nutritional requirements for many macro- and micro-nutrients, including protein, magnesium, phosphorus, potassium, zinc, selenium, vitamin A, vitamin B-12, and calcium [16]. However, there is limited research regarding the intake of dairy products and its association with the risk of PPD. An epidemiological study from Japan found an association between dairy product intake during pregnancy, including milk, yogurt, and cheese, and the development of PPD symptoms [17]. The study found that the risk of PPD symptoms is higher (9.8%) for the lowest quartile of dairy product intake than for the group with the highest intake [17]. Another study by Miyake et al. (2015) examined the intake of both dairy products and calcium on the prevalence of depressive symptoms during pregnancy [18]. It showed that calcium intake level was significantly higher in the group that scored low for depression, especially for the intake of yogurt and calcium [18]. A recent study and a systematic review [11,19] found conflicting and inconsistent associations between dairy consumption and depressive symptoms, with no conclusive evidence that individual nutrients or dietary patterns contribute to the development of perinatal depression.

LaChance et al. (2018) identified anti-depressant nutrients related to the prevention and treatment of depression [12]. These nutrients include folate, iron, omega-3 fatty acids, magnesium, potassium, selenium, thiamine, vitamin A, vitamin B6, vitamin B12, vitamin C, and zinc. However, these nutrients aligned with previous evidence suggesting that the depletion of nutrients such as folate, vitamin D, and trace minerals of calcium, iron, and zinc might be associated with the development of PPD [18,20,21]. These findings were also further emphasized by the systematic review findings of Ljungberg et al. (2020) [21].

While previous studies have examined the association between the intake of dairy products and other nutrients during pregnancy with PPD [11,17,18], there is a paucity of research as to whether the intake of dairy products and anti-depressant nutrients during the postpartum period might affect the prevalence of PPD. Therefore, the aim of this study is to assess the effects of postpartum intake of dairy products and other nutrients on PPD. We hypothesize that a higher intake of dairy products and other anti-depressant nutrients postpartum is protective against PPD symptoms. The study will add to the fundamental knowledge pertaining to dairy product intake and depression. Furthermore, the results of the study will provide specific information that may guide healthcare providers and decision makers in the development of strategies for maintaining and improving maternal mental health, and therefore women’s health outcomes and quality of life.

## 2. Materials and Methods

### 2.1. Study Design

A cross-sectional survey-based study (online questionnaire) was conducted in all regions of Saudi Arabia between February 2021 and August 2021. The study was conducted according to the guidelines proposed in the Declaration of Helsinki and was approved by the Unit of Biomedical Ethics Research Committee, Faculty of Medicine, King Abdulaziz University, Jeddah, Saudi Arabia (Registration Number: HA-02-J-008, Reference No 84-21). 

### 2.2. Study Sample 

The participants were female adults aged ≥18 and ≤50 years old, within the first year after childbirth (≥1 and ≤12 months postpartum). Those who were <18 or >50 years old, residing outside of Saudi Arabia, and/or with a history of mental illness were excluded from the study. The Epi Info online sample size calculator (Division of Health Informatics and Surveillance, and Centre for Surveillance, Epidemiology and Laboratory services, GA, USA) was employed to compute the required sample size, with a two-sided significance level (1-alpha) of 95%, a power (1-beta, % chance of detecting) of 80%, a 0.4 ratio of sample size (Unexposed/Exposed), a percent of unexposed with outcome of 15%, percent of exposed with outcome of 30%, an odds ratio of 2.43, a risk/prevalence ratio of 2.00, and a risk/prevalence difference of 15. The calculated sample size was 324 participants. By adding a 20% dropout rate, the required sample size was 388. 

### 2.3. Study Instrument and Data Collection

An electronic self-administered questionnaire was created using SurveyMonkey, incorporating a validated food frequency questionnaire (FFQ) and the Edinburgh Postnatal Depression Scale (EPDS) screening tool, with several questions on demographics, employment-related factors, family-related factors, pregnancy-related factors, health-related factors, and psychological well-being factors as previously described [22]. The survey link was disseminated using multiple social media platforms, such as WhatsApp, Twitter, and Instagram, as well as e-mail and flyers in maternity units in hospitals across the Kingdom. A reminder to participate in the study was sent on a regular basis to improve study recruitment. Participants were allowed to answer the questionnaire only once. The study protocol, procedures, and participants’ rights were explained at the beginning of the survey, and written informed consent was obtained from all participants prior to their participation. Privacy was ensured to all participants, and none of their personal information was disclosed to the public.

### 2.4. FFQ

An Arabic FFQ was adapted from the Saudi Food and Drug Administration’s FFQ [23]. One hundred and thirty-three (133) food items were included in the FFQ, using a close-ended approach. For each close-ended question, 9 answer options were provided with consumption frequency choices given as follows: never or less than once a month, 1–3 times per month, once a week, 2–4 times per week, 5–6 times per week, once a day, 2–3 times per day, 4–5 times per day, or 6+ times per day. 

An additional set of questions regarding other food items that were not listed were included as open-ended questions at the end of the FFQ to gather specific information. The FFQ also included questions about the consumption of nutritional supplements and probiotics. The nutritional values of the items were based on the Saudi Food Composition Tables for 1996 and McCance and Widdowson’s Composition of Foods Integrated Dataset for 2015 [24,25]. In this study, the FFQ was used to assess the frequency and quantity of the intake of dairy products as a food group and their composition of macro- and micro-nutrients, including protein, carbohydrates, fat, fiber, iron, potassium, thiamine, riboflavin, vitamin A (retinol & β carotene), vitamin C, phosphorus, and calcium. 

### 2.5. EPDS

The EPDS has been validated and translated into 18 languages, including an Arabic version [26]. This questionnaire is used to screen participants’ depression levels for further assessments and referrals to treatment. The EPDS is a 10-item self-report scale assessing the common symptoms of depression. Each item is scored on a four-point scale (0–3), with total scores ranging from 0 to 30 [27]. The cut-off value of ≥12 has been shown to indicate good sensitivity and specificity in Arabic-speaking cultures [28]. For the purposes of this study, an EPDS score was considered to reflect the presence of PPD at 12 or higher.

### 2.6. Statistical Analysis

All intake-related recorded data were analyzed by using Microsoft Excel. Statistical analysis was performed using IBM SPSS (version 28) statistical software. Frequency distribution was used for the demographics, eating habits, and the PPD score category (depressed/non-depressed). Mean and standard deviation were used for the summary of the PPD score and its associated items, in addition to the summary of the consumption of various dairy products and other nutrients. The median was used only for the nutritional supplement statistics. The relationships between the consumption level of various dairy products and other nutrients and PPD score were evaluated using logistic regression. A chi-square test was used to test the unadjusted association between PPD and nutritional supplements/eating habits. A *p*-value of <0.05 was considered statistically significant.

## 3. Results

### 3.1. Characteristics of Study Participants

We recorded 530 participants in the survey (Table 1). The majority of the sample (97%) were 20–39 years old with a mean age of 29.3 ± 4.9 years. The majority were Saudis (94%), non-smokers (*n* = 500, 93.8%), holders of a bachelor’s degree (*n* = 386, 69%), or had two children (*n* = 248, 46.5%). Of the participants, 28% were not working/unemployed; the rest were roughly divided between working in the public or private sector. Almost all the participants in this study were married (98%).

Almost three-quarters of the participants had had a normal birth. About half (51%) were exclusively breastfeeding, while the rest varied between those who used to but had stopped, those who were breastfeeding inconsistently or combination feeding, and those who had never breastfed (12%, 24%, and 13%, respectively). 

Most of the participants (81%) were roughly divided between those within the normal and overweight body mass index (BMI) categories; the rest were mainly obese (underweight was rare). Most participants (61%) were roughly divided between those practicing physical activity consistently and those only practicing sometimes (not regularly participating in physical activity). Lastly, around three-quarters of the participants were found to be in the range for PPD (*n* = 395, 74.11%).

### 3.2. Dairy Products and PPD

We evaluated the association between the daily consumption of dairy products and PPD. The dairy products included were milk, cheese, yogurt, and Laban (buttermilk). The daily consumption of products was divided into four levels of intake, starting from minimal intake (Q1), and ending with maximum intake (Q4) (Table 2). 

We found that the risk of depression was relatively high under the Q1 level of consumption for all five elements and that as we proceeded from Q2 to Q4, there appeared to be a smooth increment in depression risk, meaning an increase in the risk of depression as consumption increased. Therefore, both very low and very high consumption of dairy products were associated with a higher risk of depression (Table 2).

We noticed that the odds for all the elements and for all quartiles were statistically significant (*p* < 0.05) except for Laban at Q2. However, when adjusting for confounders (age, history of depression, physical activity, income, and body mass index), the previous finding (that higher consumption was associated with depression) was no longer present in dairy products (except Laban) because almost all the estimated odds were statistically insignificant. We found that the high consumption of dairy products seemed to be associated with higher risk of depression, without implying causality. Moreover, when adjusting for confounders, this association dissolved, except for Laban, where some effect can be seen (Table 2).

### 3.3. Other Nutrients and PPD

We evaluated the relationship between various other nutrients and PPD. Food elements included vitamins, minerals, and general food groups, such as fat and proteins. We found participants under Q1 had a relatively high risk of depression, as in our findings for dairy products. However, the increasing trend of depression risk from Q2 to Q4 was not very smooth. When adjusting for confounders, only Q3 for fat showed significant odds for depression (Table 3).

### 3.4. Nutritional Supplements/Eating Patterns and PPD

We also explored the association between the intake of nutritional supplements and PPD. There was a significant association between the intake of probiotics and PPD (*p* < 0.001), with a 69% rate of PPD among those taking probiotics and 90% for those not taking (Table 4). When adjusting for confounders and estimating the odds of PPD when taking probiotics, we found significant odds of PPD (estimated as 0.1; when taking probiotics, chances of PPD were 10% of the chances of not having PPD). We concluded that the chi-square association between probiotics and PPD was misleading because it showed a higher chance (90%) of PPD when using probiotics. When adjusting for confounders, we found a hidden positive effect for taking probiotics (odds for PPD of 0.1, equivalent to a probability of 9%).

## 4. Discussion

This study evaluated the intake of dairy products and other nutrients in women during the postpartum period. Our findings showed that a high consumption of dairy products and other nutrients, mainly anti-depressant ones, seems to be associated with a higher risk of PPD. However, when adjusting for confounders, this association dissolves, except for Laban intake, which shows a positive relationship with PPD. These findings differed from those of a previous study from Japan that showed no significant association between the total intake of dairy products during pregnancy and risk of PPD [29]. Another cohort study from the same authors showed that higher milk intake during pregnancy was associated with a reduced risk of PPD [17]. This inconsistency may have been due to the differences in the timing of dairy product intake during the perinatal period. The intake of dairy products was during the pregnancy period in previous studies whereas in this study the intake after childbirth (postpartum) was considered.

Our study showed no association between the intake of other nutrients—including anti-depressant nutrients—and PPD. This could be explained by the limited nutrient analysis for dairy products and foods with a cut-off of 50mg and more per serving, which may not have reflected the whole nutrient analysis per individual per day. This finding is consistent with a large cross-sectional study of 4734 participants that found no association between intake levels of calcium and depressive symptoms [30]. Results from another cross-sectional study in Japan suggested that a higher intake of magnesium, calcium, iron, and zinc had a positive effect on lowering the prevalence of depressive symptoms in Japanese employees [31]. Furthermore, previous studies have shown that the low prevalence of depressive symptoms during pregnancy was related to a higher intake of yogurt and calcium [18], which was not the case in this study.

Even though no evident relationships were noted between PPD and other anti-depressant nutrients, we did note that PPD was prevalent in women whose fat consumption is high. This could be explained by the high intake of unhealthy foods as a result of depression, as well as the changes in weight as a result of pregnancy. Evidently, the participants with a high level of depressive symptoms tended to consume more unhealthy food and had a high BMI [32]. As this study was conducted during the COVID-19 pandemic, it is also possible that the high intake of unhealthy food and the failure to follow a healthy eating pattern were attributable to the stress related to the pandemic. Recent research has shown that the prevalence of PPD was 34% during the COVID-19 pandemic [33], which is higher than the incidence reported in previous studies (10–20% pre-pandemic) [34,35,36].

With regard to nutritional supplement intake, only the consumption of probiotics showed a significant difference in the rate of PPD; 69% for those taking probiotics and 90% for those not taking it. Even when adjusting for confounders, probiotics seemed to be effective, with significant odds for PPD (estimated as 0.1; when taking probiotics, chances of PPD are 10% of the chances of no PPD). This is consistent with the results of a New Zealand study [37], in which a randomized, double-blind, placebo-controlled trial of the effect of probiotics on PPD in 423 women showed that women who received probiotics had significantly lower depression scores in the postpartum period [37].

This study has some limitations. First, the study design was cross-sectional, making it difficult to derive conclusions regarding causal links. Second, the diagnosis of PPD was established by a self-administered rating scale (EPDS) rather than by a clinician-administered structured diagnostic interview. Third, the nutrient profiling analysis was limited to dairy products and foods with a cut-off of 50mg or more of calcium per serving. Despite these limitations, to the best of our knowledge, this is the first study to examine the association between PPD and the consumption of dairy products and other anti-depressant nutrients during the postpartum period. This association offers an intriguing direction for research in alternative therapy options for PPD. Another strength of this study is the large sample size and the inclusion of participants from all regions of Saudi Arabia, increasing its potential generalizability. 

## 5. Conclusions

The results of this study suggest that the consumption of dairy products and other nutrients has no effect on PPD. The high rate of PPD among women points to the need for early PPD screening and care for women in this critical period. It also shows the importance of healthcare professionals having a better understanding of PPD and training in the use of screening tools, as well as the importance of options for referrals, therapy, and treatment beyond the standard pharmacological options. In terms of future research, large-scale studies are needed to confirm the relationship between dietary patterns and the risk of PPD, given the scarcity of evidence on this issue. In addition, it would be useful to extend the current findings by examining other lifestyle factors, such as social factors during and after pregnancy, that could be associated with PPD.

## Figures and Tables

**Table 1:** Sample Characteristics and Demographics.

Variable	Categories	*n* (%)
Age (y)	18–19	5 (0.9)
	20–29	281 (52.7)
	30–39	236 (44.3)
	40–50	11 (2.1)
Marital status	Married	523 (98.1)
	Divorced	9 (1.7)
	Widow	1 (0.2)
Nationality	Saudi	499 (93.6)
	Non-Saudi	34 (6.4)
Education	No certificate	14 (2.6)
	Secondary or below	46 (8.6)
	Diploma	35 (6.6)
	Bachelor	368 (69.0)
	Higher education	70 (13.1)
Employment status	Does not work/unemployed	149 (28.0)
	Student	32 (6.0)
	Employed (public sector)	146 (27.4)
	Employed (private sector)	176 (33.0)
	Free job	30 (5.6)
Family income (SAR)	<10,000	339 (68)
	10,000–20,000	110 (22)
	20,001–30,000	33 (7)
	>30,000	17 (3)
Region of residence	Mecca	155 (29.1)
	Breeda	75 (14.1)
	Riyadh	58 (10.9)
	Al Dammam	57 (10.7)
	Medina	44 (8.3)
	Abha	40 (7.5)
	Other	104 (19.5)
Mother language	Arabic	528 (99.1)
	English/other	5 (0.9)
No. of children	1	126 (23.6)
	2	248 (46.5)
	3	105 (19.7)
	>3	54 (10.1)
Birth type	Normal	400 (75.0)
	Cesarean birth	133 (25.0)
Breastfeeding	Yes	273 (51.2)
	Yes, but stopped	64 (12.0)
	Sometimes	126 (23.6)
	No	70 (13.1)
BMI	Underweight	16 (2.9)
	Normal	211 (38.4)
	Overweight	234 (42.5)
	Obese	89 (16.2)
Physical activity	Yes	153 (28.7)
	Sometimes	172 (32.3)
	No	208 (39.0)
Smoking	Yes	33 (6.2)
	No	500 (93.8)

Data are expressed as *n* (%). BMI, body mass index; SAR, Saudi riyal.

**Table 2:** The association between the daily consumption of dairy products and PPD.

Variable	Indicator Name	Indicator Value by Intake Level (Q)				*p*-Value
		Q1 [Lowest]	Q2	Q3	Q4 [Highest]	
Milk	Milk Intake (serv/d) ^1^	0.0	0.43	1.00	2.15	
	PPD Risk (%) ^2^	79.9	66.4	71.2	79.8	
	PPD Odds (95% CI)	1	1.97 * (1.33–2.93)	2.47 * (1.66–3.68)	3.96 * (2.5–6.25)	0.0001
	Adj. Odds (95% CI) ^3^	1	2.67 (0.31–22.73)	1.88 (0.26–13.81)	1.24 (0.19–8.34)	>0.05
Cheese	Cheese Intake (serv/d) ^1^	0.00	0.86	1.66	3.00	
	PPD Risk (%) ^2^	78.0	64.5	77.9	80.7	
	PPD Odds (95% CI)	1	1.82 * (1.26–2.63)	3.52 * (2.22–5.6)	4.18 * (2.63–6.66)	0.0001
	Adj. Odds (95% CI) ^3^	1	0.12 (0.02–1.03)	0.48 (0.04–6.19)	0.31 (0.03–3.46)	>0.05
Yogurt	Yogurt Intake (serv/d) ^1^	0.00	0.14	0.80	2.00	
	PPD Risk (%) ^2^	79.2	65.4	72.3	85.4	
	PPD Odds (95% CI)	1	1.89 * (1.32–2.7)	2.61 * (1.73–3.95)	5.85 * (3.25–10.53)	0.0001
	Adj. Odds (95% CI) ^3^	1	1.69 (0.27–10.45)	1.41 (0.17–11.64)	4.3 (0.3–62.43)	>0.05
Laban	Laban Intake (serv/d) ^1^	0.00	0.14	0.80	2.00	
	PPD Risk (%) ^2^	81.9	60.3	72.4	77.0	
	PPD Odds (95% CI)	1	1.52 (0.95–2.42)	2.62 * (1.84–3.74)	3.35 * (2.03–5.52)	0.0001
	Adj. Odds (95% CI) ^3^	1	0.05 * (0–0.93)	0.08 * (0.01–0.96)	0.15 (0.01–1.7)	0.0001
Total Dairy	Total dairy intake (serv/d) ^1^	0.86	2.14	4.01	7.09	
	PPD Risk (%) ^2^	76.7	64.3	84.7	74.6	
	PPD Odds (95% CI)	1	1.8 * (1.23–2.64)	5.53 * (3.3–9.27)	2.93 * (1.92–4.47)	0.0001
	Adj. Odds (95% CI)^3^	1	0.09 (0.01–1.07)	0.12 (0.01–1.75)	0.38 (0.03–5.39)	>0.05

^1^ Values for intake are medians. ^2^ Risk of PPD symptoms based on the EPDS for each quartile. ^3^ Adjustment for age, history of depression, physical activity, income, and body mass index. * *p* < 0.05. CI, confidence interval; PPD, postpartum depression; Q, quartile.

**Table 3:** The association between daily consumption of other nutrients and PPD.

Variable		Indicator Value by Intake Level (Q)				*p*-Value
		Q1 [Lowest]	Q2	Q3	Q4 [Highest]	
Water (mL)	Water Intake ^1^	274.58	698.65	1035.78	2242.43	
	PPD Risk (%) ^2^	76.6	69.4	77.5	71.8	
	PPD Odds (95% CI)	1	2.26 * (1.51–3.39)	3.44 * (2.2–5.37)	2.55 * (1.68–3.86)	0.0001
	Adj. Odds (95% CI) ^3^	1	0.89 (0.1–8.21)	3.41 (0.28–42.34)	0.87 (0.14–5.54)	>0.05
Fat (g)	Fat Intake	30.53	75.82	156.46	418.89	
	PPD Risk (%) ^2^	77.3	65.8	77.5	75.5	
	PPD Odds (95% CI)	1	1.92 * (1.3–2.84)	3.44 * (2.2–5.37)	3.07 * (1.99–4.75)	0.0001
	Adj. Odds (95% CI) ^3^	1	6.91 (0.53–89.82)	144.33 * (2.18–9547.67)	3.58 (0.41–31.36)	0.0001
Protein (g)	Protein Intake	22.49	54.78	102.26	225.03	
	PPD Risk (%) ^2^	77.1	66.4	72.7	78.9	
	PPD Odds (95% CI)	1	1.97 * (1.33–2.93)	2.67 * (1.75–4.06)	3.74 * (2.36–5.92)	0.0001
	Adj. Odds (95% CI) ^3^	1	0.37 (0.03–4.97)	0.57 (0.05–6.21)	5.5 (0.21–142.55)	>0.05
CHO (g)	CHO Intake	50.95	130.84	219.60	428.74	
	PPD Risk (%) ^2^	78.3	70.1	76.6	67.9	
	PPD Odds (95% CI)	1	2.34 * (1.55–3.55)	3.28 * (2.1–5.13)	2.12 * (1.41–3.18)	0.0001
	Adj. Odds (95% CI) ^3^	1	12.88 (0.85–195.97)	4.51 (0.43–47.13)	3.7 (0.4–34.01)	>0.05
Fiber (g)	Fiber Intake	1.65	7.80	16.92	40.32	
	PPD Risk (%) ^2^	87.2	70.9	64.2	71.6	
	PPD Odds (95% CI)	1	2.44 * (1.62–3.68)	1.79 * (1.21–2.66)	2.52 * (1.66–3.81)	0.0001
	Adj. Odds (95% CI) ^3^	1	0.53 (0.05–5.27)	2.04 (0.16–25.21)	1.27 (0.12–13.9)	>0.05
ASH ** (g)	ASH Intake	3.04	6.72	13.16	25.25	
	PPD Risk (%) ^2^	81.8	70.0	66.4	77.3	
	PPD Odds (95% CI)	1	2.33 * (1.55–3.51)	1.97 * (1.33–2.93)	3.4 * (2.18–5.31)	0.0001
	Adj. Odds (95% CI) ^3^	1	0.33 (0.04–2.91)	0.59 (0.05–6.94)	1.77 (0.17–18.48)	>0.05
Retinol (µg)	Retinol Intake	117.50	309.35	554.69	1252.28	
	PPD Risk (%) ^2^	76.1	70.0	73.4	77.1	
	PPD Odds (95% CI)	1	2.33 * (1.55–3.51)	2.76 * (1.8–4.22)	3.36 * (2.15–5.25)	0.0001
	Adj. Odds (95% CI) ^3^	1	0.48 (0.06–3.59)	1.5 (0.16–13.77)	1.33 (0.15–11.51)	>0.05
Β Carotene (µg)	Β Carotene Intake	174.19	620.03	1350.67	2424.24	
	PPD Risk (%) ^2^	76.9	62.0	79.6	76.6	
	PPD Odds (95% CI)	1	1.63 * (1.11–2.41)	3.91 * (2.45–6.24)	3.28 * (2.1–5.13)	0.0001
	Adj. Odds (95% CI) ^3^	1	2.17 (0.2–23.92)	0.95 (0.08–11.27)	1.09 (0.11–10.56)	>0.05
Thiamine (mg)	Thiamine Intake	0.31	0.86	1.86	4.99	
	PPD Risk (%) ^2^	75.5	69.4	72.1	78.2	
	PPD Odds (95% CI)	1	2.26 * (1.51–3.39)	2.58 * (1.7–3.91)	3.58 * (2.28–5.63)	0.00011
	Adj. Odds (95% CI) ^3^	1	1.04 (0.12–9.2)	25.49 (0.95–682.23)	2.63 (0.27–25.89)	>0.05
Riboflavin (mg)	Riboflavin Intake	0.52	1.17	2.00	4.11	
	PPD Risk (%) ^2^	76.6	66.4	76.1	75.0	
	PPD Odds (95% CI)	1	1.97 * (1.32–2.95)	3.19 * (2.05–4.96)	3 * (1.94–4.64)	0.0001
	Adj. Odds (95% CI) ^3^	1	0.65 (0.09–4.77)	1.53 (0.14–16.63)	2.35 (0.21–26.76)	>0.05
Vitamin C (mg)	Vitamin C Intake	6.09	52.00	107.54	228.33	
	PPD Risk (%) ^2^	83.3	69.4	77.8	67.3	
	PPD Odds (95% CI)	1	2.27 * (1.51–3.42)	3.5 * (2.22–5.51)	2.06 * (1.37–3.08)	0.0001
	Adj. Odds (95% CI) ^3^	1	1.24 (0.14–10.69)	28.71 (0.53–1543.57)	0.56 (0.08–3.91)	>0.05
Sodium (mg)	Sodium Intake	434.94	1440.90	2214.97	4664.47	
	PPD Risk (%) ^2^	79.6	79.6	68.5	65.7	
	PPD Odds (95% CI)	1	3.91 * (2.45–6.24)	2.18 * (1.45–3.27)	1.92 * (1.29–2.86)	0.000
	Adj. Odds (95% CI) ^3^	1	2.72 (0.33–22.3)	1.64 (0.16–17.35)	2.08 (0.29–15.15)	>0.05
Potassium (mg)	Potassium Intake	777.55	2055.05	4053.13	8410.87	
	PPD Risk (%) ^2^	82.7	77.5	62.7	72.7	
	PPD Odds (95% CI)	1	3.44 * (2.2–5.37)	1.68 * (1.14–2.48)	2.67 * (1.75–4.06)	0.000
	Adj. Odds (95% CI) ^3^	1	1.35 (0.16–11.12)	1.89 (0.2–18.22)	2.01 (0.28–14.52)	>0.05
Calcium (mg)	Calcium Intake	372.04	835.19	1419.41	2944.36	
	PPD Risk (%) ^2^	78.2	66.7	75.5	75.5	
	PPD Odds (95% CI)	1	2 * (1.35–2.97)	3.07 * (1.99–4.75)	3.07 * (1.99–4.75)	0.0001
	Adj. Odds (95% CI) ^3^	1	0.7 (0.09–5.51)	1.37 (0.17–11.16)	1.16 (0.14–9.88)	>0.05
Phosphorus (mg)	Phosphorus Intake	477.06	1133.37	2145.35	4627.63	
	PPD Risk (%) ^2^	79.3	70.3	68.5	77.5	
	PPD Odds (95% CI)	1	2.36 * (1.57–3.55)	2.17 * (1.46–3.24)	3.44 * (2.2–5.37)	0.000
	Adj. Odds (95% CI) ^3^	1	0.65 (0.06–7.72)	0.74 (0.06–8.61)	9.3 (0.38–225.88)	>0.05
Iron (mg)	Iron Intake	3.46	14.91	29.75	60.09	
	PPD Risk (%) ^2^	84.1	75.7	66.4	68.9	
	PPD Odds (95% CI)	1	3.12 * (2–4.85)	1.97 * (1.32–2.95)	2.21 * (1.47–3.34)	0.0001
	Adj. Odds (95% CI) ^3^	1	1.04 (0.12–8.78)	2.97 (0.2–43.51)	1.32 (0.15–11.53)	>0.05

^1^ Values for intake are medians. ^2^ Risk of PPD symptoms based on the EPDS for each quartile. ^3^ Adjustment for age, history of depression, physical activity, income, and body mass index. * *p* < 0.05. ** ASH refers to minerals. CI, confidence interval; PPD, postpartum depression; Q, quartile.

**Table 4:** The association between the intake of nutritional supplements/eating patterns and PPD.

Nutritional Supplements/Eating Patterns	Chance of PPD (Not Following)	Chance of PPD (Following)	Chi-Square *p*-Value	Odds of PPD with Nutritional Supplements/Eating Patterns
Vitamin D	74.2%	75.5%	0.756	0.64 (0.14–2.94)
Calcium	73.4%	74.4%	0.836	0.89 (0.17–4.74)
Omega 3	71.4%	78.4%	0.093	1.15 (0.2–6.79)
Probiotics	69.0%	90.2%	<0.001	0.1 * (0.01–0.93)
Fiber	75.6%	71.8%	0.364	1.17 (0.28–4.90)
3 meals a day	71.1%	76.3%	0.194	0.62 (0.14–2.79)
Snacks in between	66.1%	77.7%	0.005	2.58 (0.51–13.08)
Taking specific Laban brands (Activia, Danone, Kefir)	72.0%	77.4%	0.188	0.92 (0.23–3.63)

*p < 0.05.

## Data Availability

All data results are presented as texts and in tables of this article. The data is not publicly available because further research is being done and more manuscripts are being prepared. Data for the current study will be available upon reasonable request from the principal investigator or corresponding author.
